# Stakeholder selected strategies for obesity prevention in childcare: results from a small-scale cluster randomized hybrid type III trial

**DOI:** 10.1186/s13012-021-01119-x

**Published:** 2021-05-01

**Authors:** Taren Swindle, Nicole M. McBride, James P. Selig, Susan L. Johnson, Leanne Whiteside-Mansell, Janna Martin, Audra Staley, Geoffrey M. Curran

**Affiliations:** 1 Department of Family and Preventive Medicine, University of Arkansas for Medical Sciences, 4301 W. Markham St, #530, Little Rock, AR 72205-7199 USA; 2 Embedded Preventive Behavioral Health Capability, Marine Corps Community Services, III MEF, United State Marine Corps, Okinawa, Japan; 3 College of Public Health, Department of Biostatistics, University of Arkansas for Medical Sciences, 4301 W. Markham St., #781, Little Rock, AR 72205 USA; 4 Department of Pediatrics, University of Colorado Anschutz Medical Campus, 12700 East 19th Avenue Box C225, Aurora, CO 80045 USA; 5 College of Medicine, University of Arkansas for Medical Sciences, 4301 W. Markham St, #530, Little Rock, AR 72205-7199 USA; 6 Department of Pharmacy Practice and Psychiatry, University of Arkansas for Medical Sciences, 4301 W. Markham St, #522-4, Little Rock, AR 72205-7199 USA; 7 Central Arkansas Veterans Healthcare System, North Little Rock, AR 72114 USA

**Keywords:** Implementation strategies, Childcare, Obesity prevention, Implementation science, Early care and education, Nutrition

## Abstract

**Background:**

Together, We Inspire Smart Eating (WISE) is an intervention for the early care and education setting to support children’s exposure to and intake of fruits and vegetables. WISE emphasizes 4 evidence-based practices (EBPs): (1) use of a mascot; (2) educators’ role modeling; (3) positive feeding practices; and (4) hands-on exposures. The current study reports on a small-scale implementation trial aimed at improving the use of WISE EBPs by teachers.

**Methods:**

A Hybrid Type III Cluster Randomized Design compared a Basic and Enhanced implementation strategy. The Basic Strategy included training and reminders only; the Enhanced strategy was a multi-faceted package of stakeholder-selected strategies including a leadership commitment, an implementation blueprint, a local champion, an environmental reminder of the EBPs, facilitation, and tailored educational resources and incentives. All study sites were Head Starts. Sites were randomized using a balancing technique that considered site characteristics; 4 sites (20 classrooms, 39 educators, 305 children) received Enhanced support; 5 sites (18 classrooms, 36 educators, 316 children) received Basic support. RE-AIM guided the evaluation, and implementation fidelity was the primary outcome. Strategies were assessed using examination of data distributions and unadjusted comparisons (*t* tests) as well as general linear and mixed effects models controlling for covariates.

**Results:**

For the primary outcome of fidelity, the Enhanced group had significantly higher means for 3 of 4 EBPs. Multivariate models explained a significant portion of variance for both mascot use and hands-on exposure with a significant positive effect observed for treatment condition. The Enhanced group also had higher rates of Appropriateness and Organizational Readiness for Implementing Change (as indicators of implementation and adoption, respectively). There was no significant difference between groups for indicators of Reach, Effectiveness or Maintenance. Formative interviews indicated key targets for iteration and potential mechanisms. Key events were catalogued to provide context for interpretation (e.g., 61% of classrooms with turnover).

**Conclusions:**

Findings were mixed but suggested promise for the Enhanced strategy, especially considering key events of the study. Implementation fidelity improvements occurred mainly in the last 3 months of the school year; additional time may be needed to translate to improvements in child outcomes.

**Trial registration:**

NCT03075085 Registered 20 February 2017.

**Supplementary Information:**

The online version contains supplementary material available at 10.1186/s13012-021-01119-x.

Contributions to the literature

- This implementation study illustrates a mixed method evaluation with a small-scale cluster randomized Hybrid Type III trial to assess stakeholder-selected implementation strategies in a community setting.
- This study provides an example of rigorous tracking and reporting of a package of multi-faceted implementation strategies, including facilitation, during the course of an implementation trial in the community.
- Reporting of results includes a log of key events influencing the process and findings of our trial, which may provide a model for other studies to illustrate key contextual events surrounding the research process.

## Background

The rate of overweight and obesity in children between the ages of 2 and 5 years is 16% with significant increases in obesity and severe obesity in recent years [1]. Given that early life obesity predicts weight trajectory and health outcomes in later life [2–4], children need effective prevention and intervention efforts to reduce excess weight. One potential target of prevention efforts is children’s dietary habits. Diet quality is associated with weight status among children [5], even after adjusting for physical activity [6]. Thus, poor quality diet is a key preventable predictor of excess weight.

The American Academy of Nutrition and Dietetics outlines research-based benchmarks for the early care and education setting (ECE, i.e., childcare) to meet children’s dietary needs and provide an environment for healthy growth [7, 8]. These include providing healthy foods, respecting child hunger and satiety, encouraging role modeling by educators, providing nutrition education, and providing training for educators. Implementation of these best practices in the early care and education environment has the potential for significant positive impact. For example, a 2016 systematic review by Sisson and colleagues found that interventions in the ECE setting frequently have desired effects on obesity (48%) and obesity-related outcomes (physical activity, 73%; diet, 87%; screen time, 63%) [9]. A more recent umbrella review of 12 systematic reviews on the effects of ECE-based interventions found significant influences on children’s dietary habits [10]. The strongest interventions on Obesity-related outcomes were those that addressed multiple components (e.g., child behavior, educator behavior, center policy) and engaged parents. These findings support that the ECE setting is an important context for implementation of evidence-based practices and programs to improve children’s diets and reduce risk for obesity.

Together, We Inspire Smart Eating (WISE) is an intervention designed for delivery in the ECE setting to support children’s exposure to and intake of fruits and vegetables and to promote the development of self-regulation for food intake. Prior research on WISE has documented positive impacts on educator knowledge and children’s intake of target foods [11–13]. However, the prior research also provides evidence of barriers that educators experience in attempts to implement the evidence-based practices that comprise WISE [14]. Challenges in implementing WISE are not unique. For example, a review of 18 studies in ECE settings found that educators often do not follow evidence-based practices that include identifying hunger cues, avoiding the use of foods for celebration/reward, and allowing children to decide how much to eat without pressure [15]. Active efforts are needed to close these implementation gaps.

A few studies have focused on promoting nutrition and preventing obesity in early childhood settings through the application of Implementation Science. To date, these studies, primarily in Australia, have deployed rigorous randomized designs to compare strategy packages to implementation as usual (i.e., no implementation strategies) [16–20]. Strategy packages in these studies have included 5–9 discrete strategies including various combinations of supporting leadership, training staff, providing implementation staff, academic detailing, audit and feedback, resource materials, communication/marketing plans, and incentives/recognition. By and large, the results have supported the effectiveness of multi-faceted approaches for successful implementation of policies and environmental changes that support healthy eating [17–20]. Singular implementation strategies (i.e., education, serial audit and feedback), however, have not outperformed control conditions at increasing implementation outcomes in early childhood settings [21, 22]. Primarily, these studies have focused on supporting uptake of policy or guidelines and its implementation by center-level leadership (e.g., written physical activity/nutrition policies (PA) [16, 19, 22], restriction of screen time [16, 22], provision of only water/reduced fat milk [16, 19, 22], provision of adult-guided movement/motor skill development [16, 22], monitoring of parent-packed lunches [16, 19], healthy school meal policies [17, 20], provision of fruit and vegetable breaks [18], provision of staff training in nutrition [19], provision of nutrition programs/resources for parents [19, 22], provision of healthy foods to children [19, 20], provision of learning about healthy foods, [22]). Less research has targeted implementation strategies aimed at improving educator’s use of evidence-based practices. Although educator behaviors such as role modeling healthy eating/PA [16] and encouragement of healthy eating/PA [16] were targeted in one study, these outcomes were combined with center-level policy uptake when examining effectiveness of the implementation strategies.

Systematic reviews of implementation trials to improve policies and practices related to obesity prevention and nutrition and physical activity promotion have identified a focus on educational strategies in both ECE [23] and school settings [24]. Further, educational strategies have had inconsistent effects on targeted implementation outcomes. Researchers have less frequently studied more comprehensive strategy packages that address multiple levels of implementation. The systematic review of ECE studies identified no studies examining costs of implementation strategies [23]. Examination of multi-level, multi-faceted strategies and documentation of their costs could have potential to advance the field.

The current study seeks to report on a small-scale implementation trial aimed at improving the use of the evidence-based practices of the WISE curriculum by early care and education teachers in the southern United States [25]. Specifically, after a rigorous process of partnership with stakeholders to select and tailor strategies, this study compared a “Basic” implementation approach (i.e., training and reminders only) to an “Enhanced” implementation approach including 8 discrete implementation strategies, tailored to individual contexts and educators (e.g., individualized incentives and resources, targeted facilitation). Throughout the study, we collected formative data from interviews with educators about their experiences in implementation and perceptions of implementation strategies. These data were used in collaboration with stakeholders to inform adjustments to the strategies both during and after the study. The study took the form of a small-scale randomized implementation trial [26] with the primary goal of examining preliminary effects on implementation outcomes.

## Methods

### Study design

**Fig. 1:**
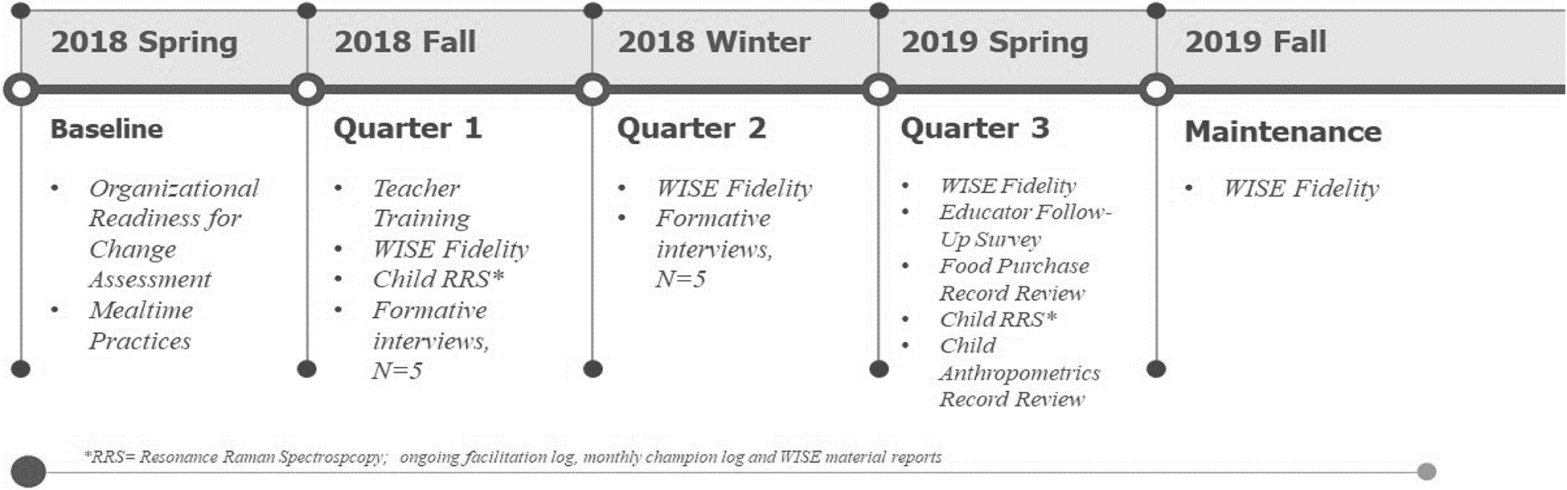
Timeline of project events

This study used a small-scale Hybrid Type III Cluster Randomized Design with the goal of comparing a Basic and Enhanced multi-faceted implementation strategy (NCT03075085, see Table 1). Hybrid Type III trials focus primarily on assessing the value of an implementation strategy or package of strategies for achieving improved implementation of evidence-based practices with a secondary focus on assessing effects on targeted health outcomes [27]. Our study is a small-scale implementation trial, defined in this way given the inclusion of only one ECE agency with 9 sites in a single county in one southern state as well as ongoing improvements to the implementation strategy based on formative evaluation. In ECE, a childcare site is a physical location serving multiple classrooms, typically with 2 educators per classroom and a director providing leadership to the site. In our study, all sites were under the administration of one centralized agency. A childcare site was deemed as a cluster for randomization, and all 9 sites of the participating agency were randomized to receive either Basic or Enhanced support. In total, 4 sites, 20 classrooms, 39 educators, and 305 children received Enhanced support; 5 sites, 18 classrooms, 36 educators, and 316 children received Basic support. Our full protocol [28] and developmental evaluation are described in detail elsewhere [14]. In addition to monitoring implementation and child health outcomes, we collected formative and summative evaluation data to assess the feasibility, acceptability, and fidelity of the Enhanced WISE strategies during real-time implementation [29] and to guide revisions of the implementation strategies *during the study* [27]. Specifically, formative interviews with teachers were collected at the end of the fall and winter quarters of the school year (2018–2019). All study activities were reviewed and approved by the IRB at the University of Arkansas for Medical Sciences. The timeline of project events is presented in Fig. 1.

**Table 1:** Hybrid III Implementation Trial: outcomes by RE-AIM construct

Construct	Outcomes	Level of measurement	Data Source
*R*each	• Number of lessons delivered to children • Number of resources distributed	Cluster	• Monthly report from teachers
*E*ffectiveness	• Child body mass index • Child dietary intake	Individual	• Review of agency records • Child resonance Raman spectroscopy scan
*A*doption	• Number of WISE lessons prepared; • Organizational readiness for implementing change score	Cluster; individual	• Review of agency food purchase records • End of year teacher survey
*I*mplementation	• WISE fidelity scores for 4 evidence-based practices • Acceptability, Feasibility, Appropriateness scores	Individual	• Observed wise fidelity • End of year teacher survey and formative interviews
*M*aintenance	• WISE fidelity scores in following school year	Cluster	• Observed WISE fidelity

#### Study participants

This project took place in a southern state within the context of an urban (population = 197, 881) federally funded Head Start program. Head Start provides services and education to children in families experiencing poverty. For example, a family of 4 qualifies for Head Start services in this state in 2020 if the household income is less than $26,200. The project informed all parents of the purpose of the study, and parents indicated if their children could participate in assessments and review of records for relevant information. Educators participating in interviews provided verbal consent, and data collection activities in the classroom were deemed consistent with usual educational practice by the IRB.

### Intervention

WISE is a nutrition promotion and obesity prevention program designed to increase self-regulation and consumption of fruits and vegetables [11–13]. WISE is designed specifically to be used in early care and education centers. It utilizes 3 components for its structure: (a) a curriculum for the classroom, (b) a training for educators to implement the curriculum, and (c) a system to educate parents on WISE concepts. Lessons from the WISE program are taught on a weekly basis in classrooms and focus on introducing eight designated fruits and vegetables to the children. WISE emphasizes 4 key, evidence-based practices: (1) use of a mascot (Windy Wise, an owl) to give children a friendly character to associate with healthy eating; (2) educators’ role modeling positive food interactions for children to observe; (3) educators’ positive feeding practices to encourage children’s individual self-regulation and autonomy in eating; and (4) implementing hands-on, sensory experiences with target fruits and vegetables (called “food experiences”) to increase awareness and acceptance of healthy foods. Previous research of WISE has shown positive impacts on children’s consumption of target fruits and vegetables [12]. Additional detail can be found elsewhere [11, 14].

### Implementation strategy selection and tailoring

We developed a multi-faceted implementation strategy by partnering with stakeholders through application of Evidence-Based Quality Improvement Panels (EBQI) that was delivered subsequently to the Enhanced condition. EBQI is a flexible process conducted across a series of meetings with topic-driven agendas [30]. The EBQI panel consisted of Head Start educators (*N* = 7), directors (*N* = 2), and a cook (*N* = 1), along with parents of children (*N* = 2) in the Head Start program. To recruit stakeholders, the research team shared information on the study with sites and invited volunteers. Directors and educators volunteered based on these invitations. Directors nominated parents for the panel, suggesting parents they believed would be active and engaged participants. Directors collected consent to contact from parents, and the research team contacted the parents to discuss participation. Two meetings were dedicated to each WISE practice. First, we used structured activities [31] (e.g., card sort) to help guide stakeholders in prioritizing barriers and facilitators gathered in our prior qualitative work for each practice. Then, the research team mapped prioritized barriers and facilitators to implementation strategy options from the Expert Recommendations for Implementing Change [32]. In a second session, to reach a consensus on the multi-faceted implementation strategy package, we used techniques from concept mapping [33] whereby stakeholders voted on the potential importance and feasibility of each potential strategy. We mapped rates of importance and feasibility onto “GoZone” plots to facilitate easy visualization of the ratings and subsequent discussion of educator preferences and prompt operationalization of their choices. The research team developed draft plans and materials based on these choices. Finally, the research team presented the draft strategies/tools, collected feedback for revisions, and received final approval to test them. Table 2 presents the specification of the resulting strategies consistent with recommendations from Proctor et al. [44] in implementation strategy reporting. The strategies selected included obtaining a formal leadership commitment, developing an implementation blueprint, identifying and preparing a local champion, providing a reminder of the evidence-based practices on a cutting board for WISE lessons, delivering facilitation to support the context and educators in implementation, and distributing both educational resources and incentives based on educators’ implementation behaviors.

**Table 2:** Specification of WISE implementation strategies from a Hybrid III Implementation Trial

Strategy	Actor(s)	Action	Temporality	Dose	Justification
Obtain formal commitments	WISE facilitators and directors	Sign commitment after facilitators and directors discuss	Before teacher training	1-time	Address barriers of awareness and leadership buy-in
Identify and prepare champions [34, 35]	Appointed champion at each site	Provide 2-h training on advocating and navigating for WISE	Within 2 months of teacher training	1 training: monthly contacts and as requested	Address barrier of capacity for change
Develop an implementation blueprint [36]	WISE staff review with directors	Provide target milestones for each phase and testimonials	With director before training	As desired	Support integration of change and leadership engagement
Remind educators [37, 38]	Classroom cutting board	Provide visual reminder of EBPs	At WISE lessons	As desired	Provide timely reminders
Implementation facilitation [39]	WISE facilitators	Provide in-person visits to directors, champions, and educators	Beginning 2 weeks after training; continuing for 1 year	Twice monthly; more upon request	Address contextual barriers; task and goal support; build and leverage relationships; holistic/enabling focus [40, 41]
Distribute educational materials [37, 38, 40–42]	WISE facilitators and champions	Provide educators with tailored educational material on the use of EBPs	Each quarter	Varies by teacher (up to 8 resources given)	Address barriers of beliefs, knowledge and skills; give advantages of EBPs.
Provide incentives [43]	WISE facilitators and champions	Provide incentives to educators for use of EBPs	Each quarter	Varies by teacher (up to 8 earned)	Leverage norms to increase use of EBPs; increase adoption

### Facilitation

External facilitation by WISE staff was the implementation strategy selected by the EBQI as critical to the foundation of our approach. For this project, 3 individuals knowledgeable of the ECE context and WISE intervention acted as facilitators. All facilitators were employees of the research university; one was the study principal investigator (PI), and 2 were research assistants (RA). The PI had experience as an early educator and professional training in child development, psychology, and implementation science. One RA had over 20 years in the field as an educator and professional training in education; the second RA had over 5 years working with the WISE intervention and experience in community development. The study PI and the second RA completed the Veteran’s Affairs Quality Enhancement Research Initiative (VA QUERI) Implementation Facilitation Training [45]. This training informed the strategy and activities of the facilitators; all facilitators reviewed this manual together prior to facilitation and built consensus around operationalizing facilitation practice in the childcare setting. Key aspects of facilitation (e.g., framing to educators, interface with champion, desired frequency) were discussed and refined with the EBQI panel. Fidelity data collection, formative interviews, and EBQI feedback guided the facilitation strategy. Facilitators prioritized working with classrooms with the lowest fidelity to address barriers, assess support needs, and build relationships with those experiencing implementation challenges. Facilitators did not participate in data collection.

### Randomization

The biostatistician, who was blinded to sites, randomized sites to conditions using tools from the randomizeR package in R software [46]. First, the software generated all possible assignments of the 9 sites to the 2 conditions. Second, this set of combinations was narrowed to those with a similar number of sites in each condition (i.e., n_Basic_ = 5 and n_Enhanced_ = 4). Third, key characteristics of the sites were considered including site-level average scores on the Organizational Readiness for Change Assessment (ORCA), number of classrooms, presence of a stakeholder from the EBQI panel, use of family style meals (yes/no), location of lunch meal (classroom/lunchroom), and site-level average scores on existing educator mealtime practices (both supportive and unsupportive). These characteristics were collected through a pre-implementation educator survey and considered for balancing randomization given the variability among sites in characteristics relative to this specific intervention (e.g., existing nutrition practices) and to implementation more generally (e.g., implementation climate). Fourth, MANOVA was used to compare treatment groups for all possible group assignments in step 2. Random assignments that produced groups similar on the vector of covariates (i.e., MANOVA *F* statistic < 3) with a difference of five or fewer in the number of classrooms in each condition were retained. This yielded 130 possible random assignments. Finally, the biostatistician used the random sample tool to select one combination from the 130 remaining combinations.

### Measures

Table 1 summarizes the measures for the study, how they align with the RE-AIM framework [47] (which guided our selection of measures), and their source of data collection. Implementation measures assessed Reach, Adoption, Implementation, and Maintenance. Effectiveness is reflected by child outcome measures. Consistent with our Hybrid III design, Implementation was the primary outcome; other outcomes were of secondary interest.

### Reach

#### Number of lessons delivered

At the end of each month, research staff asked educators to complete a one-page survey that indicated the number of WISE lessons they delivered for the month, WISE practices they used in the classroom, and their use of implementation support materials/resources (in the Enhanced group only). The proportion of the target number of lessons delivered across the school year was calculated based on these self-reports to get an assessment of Reach. A teacher could choose not to deliver a WISE lesson even if her site prepared and provided the supplies.

#### Number of resources distributed

On their monthly survey, teachers reported on the frequency of their delivery of resources to parents (e.g., WISE recipes, farmer letters). These were summed for the month to create a total number of resources distributed.

### Effectiveness

#### Child anthropometrics

As part of Head Start record keeping, children’s height and weight is measured twice per year. These measurements were made in collaboration with a University nutrition and dietetics department. Undergraduate and graduate students, trained and overseen by a professor (PhD) and a registered dietician obtained the data near the beginning and end of the school year. Height, weight, date of birth, date of measurement, and child gender were used to calculate BMI-for-age percentile (body mass index), consistent with the US Centers for Disease Control growth charts [48].

#### Resonance Raman spectroscopy

Resonance Raman spectroscopy (RRS) technology [49–51] provided assessment of consumption of fruits and vegetables by children. This non-invasive tool scans the palm of the hand to detect skin carotenoids, which indicates consumption of colorful, carotenoid-rich fruits and vegetables. Skin carotenoid levels correlate strongly with levels in blood and tissue cells with higher scores reflecting higher carotenoid intake. Scarmo and colleagues validated RRS’ accuracy with preschool children [39].

### Adoption

#### Number of lessons prepared

The Head Start registered dietitian provided food purchase records for all sites across the school year. WISE staff reviewed all food purchase records to identify purchase of WISE foods and then logged these into a database to record the amount and site of purchase. The total servings purchased were compared to the target for the servings necessary to do a weekly WISE lesson as an indicator of adoption.

#### Organizational readiness for implementing change

At the end of the school year, educators completed a comprehensive survey about their experiences with WISE and its implementation as well as their school year. This survey included a subset of items (*n* = 5) from the Organizational Readiness for Implementing Change (ORIC) measure [52]. Scale score were created by averaging items; higher scores reflect greater readiness.

### Implementation

#### WISE fidelity

The WISE Fidelity measure was created consistent with recommendations from Schoenwald and colleagues [53] on fidelity measurement, and details are published elsewhere [54]. In brief, fidelity scores are created by averaging relevant items for each WISE EBP; higher scores reflect greater fidelity. Trained observers visited classrooms quarterly to record fidelity to WISE via a 26-item document. The trained observers (*N* = 11) consisted of undergraduate students in the fields of sociology and child development, graduate students in the fields of nutrition and psychology, and working professionals trained in education and public health. A 4-h, in-person observer training focused on (a) each item’s purpose, supported by examples; (b) examining fidelity ratings; and (c) subtle integration and observation within the classroom. Videos were used for observers to practice coding the forms, initially with the aid of a gold-standard observer and then independently. Prior to field observation, observers attained an inter-rater reliability of at least 85% on two occasions. Upon reaching reliability with video, observers were required to demonstrate reliability in a project classroom with a gold-standard observer. Observations occurred in every classroom once per quarter (i.e., Sept–Nov, Dec–Feb, March–May).

#### Acceptability, appropriateness, and feasibility

The end of year teacher survey also included adapted questions from Weiner et al. [55] to assess Acceptability, Appropriateness, Feasibility of WISE as an indicator of Implementation. Scale scores were created for each indicator by averaging relevant items; higher scores reflect greater perceived acceptability, appropriateness, and feasibility.

### Maintenance

#### WISE fidelity in following school year

In the fall of the following school year, the research team observed WISE fidelity for teachers at a classroom lesson to determine maintenance of EBP use.

### Process and formative evaluation measures

Process evaluation data were used to document the study activities including the activities of both the facilitator and the champion. Formative evaluation is a “rigorous assessment process designed to identify potential and actual influences on the progress and effectiveness of implementation efforts” [29] that is conducted within the timeline of a study and used during and/or after the study to make iterations. We collected formative interviews with educators two times—at the end of the 1st and 2nd quarters of the school year (November and Feb). Administrators and champions provided interviews at the end of the study.

#### Facilitation log

Facilitators logged all activities in a REDCap [56] database on a mobile phone application hosted at the University of Arkansas for Medical Sciences (https://base.uams.edu/redcap/surveys/?s=T8RYRDRLFY). REDCap (Research Electronic Data Capture) is a secure, web-based software platform designed to support data capture for research studies. The log captured date, facilitator name, site, start and end time, estimated travel time, event type, mode of communication, personnel contact, primary and secondary activities, follow-up needed, as well as any resource delivery. Facilitators indicated the event type as one of six options: in-person, prep time, one-on-one meeting, site visit, meal, or WISE lesson observation. Mode of communication captured whether the contact was in-person, by phone, or email. Facilitators marked all personnel they had contact with while at each site. The list included Directors, Site Champions, Lead and Assistant Educators, Family Service Coordinators, Admin personnel, Clerical Support, and Site Cooks. Facilitation activities, drawn from the VA QUERI manual and redefined for our study, included Assessment, Program Adaptation, Problem Identification/Problem Solving, Program Marketing, Stakeholder Engagement, Audit and Feedback, Network Development, and Education. The facilitator would also select the primary activity for each visit. If follow-up was needed, notes were added for the follow-up. These logs informed estimation of costs.

#### Champion log

Once per month, research staff requested that champion educators complete a short survey asking about activities over the prior month. This brief survey asked champions to estimate how much time they spent with champion specific activities, how many fellow educators they supported, and how many resources/incentives they delivered.

#### Semi-structured formative interviews

At the end of the fall and winter quarter of the school year, the analyst identified a pool of educators with the greatest fidelity shortfalls (not achieving fidelity in 3 or more of the 4 WISE EBPs). Five educators from this pool were selected randomly for semi-structured interviews at the end of the fall and winter quarters. Site leadership and champions were interviewed at the end of the spring quarter. The interview guide was informed by i-PARIHS and focused on identifying barriers and facilitators to the use of WISE (i.e., innovation), barriers and facilitators they were experiencing in their center around WISE implementation (i.e., context), and perceptions of each of the eight implementation strategies (i.e., facilitation). The interviews were collected by the PI (TS) and one research assistant (KD).

### Data analysis

Given the size of our sample (less than 10 per cluster) and small-scale nature of the trial (e.g., single agency, single state), we focused on unadjusted comparisons (*t* tests) and examination of data distributions to assess the implementation strategies. Specifically, educator and classroom level outcomes were examined using *t* tests. To explore adjusted group differences and inform future studies, we also examined group differences using general linear and mixed effects models controlling for covariates, which are reported in Supplementary File 1 due to the under-powered nature of these analyses. The sample size and related power for child level outcomes also allowed mixed effects general linear models to account for nesting of children within classrooms. Approximately 2% of youth at both RRS data collection time point yielded values < 3000 [57], and these values were treated as missing due to the unreliability of estimates in this range. Only youth with pre- and post-data for Effectiveness outcomes (RRS and BMI) were retained in respective analyses. Youth with less than 5 months between anthropometric assessments were not included (*n* = 34) to limit variability between measurement occasions. All available cases were used in the remainder of analyses.

Qualitative analyses of interviews were pragmatic and deductive [58], reflecting a directed content analysis approach [59]. Specifically, the research team focused on identifying barriers and facilitators for the i-PARIHS constructs of innovation and context as well strengths and weakness of each implementation strategy. Three coders (the TS, JM, and MD) completed analysis of interviews. First, all 3 coders coded one interview together as a group and came to consensus on coding. Then, the coders coded an additional two interviews individually before coming together to compare codes, refine the start list into a codebook with examples and define decision rules (e.g., quotes may be coded with multiple codes). Next, JM and MD coded 3 interviews and compared their coding to ensure shared understanding of the codebook; the TS resolved disagreements when consensus was not present and helped to specify the codebook further when needed. Finally, MD coded the remaining 8 interviews, consulting JM and the PI on codes that were not clear (Table 3).

**Table 3:** Child level and teacher level characteristics across each treatment condition and total sample

		Basic (*N* = 210)	Enhanced (*N* = 203)	Test statistic (*t* or *X*^2^)	Study total (*N* = 413)	Program served^b^
Child	Female, %	62.7	52.9	3.78*	58.1	
	Age, years *M* (SD)	4.1 (0.6)	4.1 (0.6)	0.24	4.1 (0.6)	
	Latino/a, %	40.8	20.8	16.16***	29.5	22.4%
	White, %	11.3	15.6	0.85	12.4	5.4%
	Black, %	60.6	75.0	8.21**	69.2	73.1%
	Parent no high school degree, %	28.7	13.3	12.92***	19.9	31.4%
	Fall 2018 RRS, *M* (SD)	25403.9 (9032.6)	24732.6 (9041.3)	0.73	25540.6 (9281.98)	
	Spring 2019 RRS, *M* (SD)	29209.1 (10141.55)	28045.5 (9044.17)	1.27	29303.1 (9388.11)	
Teacher		*N* = 36	*N* = 39		*N* = 75	
	Female,^a^ %	100	100		100	
	Race			2.47		
	White, %	22.2	10.5		15.8	
	Black, %	69.4	84.2		77.6	
	Other, %	8.4	5.2		6.5	
	Ethnicity			.007		
	Latina, %	5.6	5.1		5.2	
	Age, %			5.12		
	19–24 years	2.8	17.9		10.3	
	25–34 years	25	15.4		19.5	
	35–40 years	13.9	10.3		11.7	
	41+ years	58.3	56.4		58.4	
	Education			4.05		
	HS/ HS+ some college	27.8	41.0		35.1	
	Associate’s	33.3	35.9		35.1	
	Bachelor’s degree or more	38.9	23.1		29.9	
	Teaching experience, %			5.17		
	< 1 year	0	12.8		6.5	
	1–10 years	33.3	33.3		32.5	
	11–20 years	30.6	23.1		26.0	
	21+ years	36.1	30.8		35.1	

^a^All teachers were female

^b^Based on publicly available data from: https://arheadstart.org/index.php?option=com_content&view=article&id=96&Itemid=125https://arheadstart.org/index.php?option=com_content&view=article&id=96&Itemid=125

*p ≤ .05; **p < .01; ***p < .001

## Results

### Sample characteristics

Table 4 presents demographic information for the educators and children in this study by treatment condition at baseline. There were no differences between the Basic and Enhanced groups on educator characteristics. Parent education and racial differences were noted between the Basic and Enhanced groups with the Basic group having a greater proportion of Latinx children, fewer Black children, and a greater proportion of parents with no high school degree. Statistical test values and significances are provided in Table 4 for the subset of children with parental permission and full demographic data. Of the children included in our study compared to the children served by the program as a whole, 7% more were White; and about 7% fewer were Latinx.

**Table 4:** Results summary by RE-AIM construct

Construct	Results summary
*R*each	The number of lessons was 5% higher in Enhanced sites, but this was not a significant difference. There was no difference between conditions in the number of resources distributed to parents by educators.
*E*ffectiveness	Both groups experienced significant improvements in RRS; there was no difference between groups. There was no change in BMI across the school year for either treatment condition.
*A*doption	The agency purchased 84% of the target servings of foods needed to fully deliver WISE. The Enhanced group had significantly higher Organizational Readiness for Implementing Change.
*I*mplementation	Of 4 evidence-based practices, the Enhanced group had significantly higher means for 3 practices (mascot use, role modeling, and hands-on exposure). The Enhanced group had higher means for appropriateness, acceptability and feasibility. This was a significant difference for the outcome of appropriateness but not acceptability or feasibility.
*M*aintenance	There was no difference in maintenance of fidelity between conditions for the 19 teachers that remained.

#### Delivery of enhanced implementation intervention

Of the 20 classrooms who received Enhanced support, 18 completed a full school year, and 2 were added mid-year. All directors at the Enhanced sites took part in a pre-implementation meeting with a WISE facilitator in which the formal commitment was discussed and signed and the implementation blueprint reviewed. Each Enhanced site designated a Champion who received a 2-h training in navigating challenges, educating their peers, and advocating for WISE at their center.

Delivery of facilitation was consistent with planned levels. On average, each site was visited 19 times across the school year with an average of 4 visits per classroom. Each visit lasted, on average, 30 min. The top activities were Stakeholder Engagement and Education. Educational resources were delivered systematically at the end of the Fall and Winter quarters and on an as needed basis in between. In total, 46 educational handouts were distributed across classrooms at Enhanced sites. On average, each classroom received 2.6 handouts (Min = 0, Max = 7). Brief educational videos to reinforce the WISE components (*N* = 37) were distributed across classrooms at Enhanced sites, an average of two video resources per classroom (Min = 1, Max = 5). Incentives were delivered at the end of each quarter. There were 79 incentives given in total, 4 on average per classroom (Min = 1, Max = 11).

Costs of both the Basic and Enhanced multi-faceted strategy were estimated. The Basic strategy costs totaled $640, which included the cost of WISE training ($400), and 10 h of an RA’s time to create the WISE Newsletters ($23.88/h salary and benefits). This cost corresponds to $35 per classroom in the Basic condition. The Enhanced multi-faceted strategy estimated costs totaled $5218, which included salary and fringe benefits for 3 facilitators, allowable mileage reimbursements at a rate of $0.42/mile for travel expenses, and the actual cost of incentives and resources. The facilitator salaries included that of the PI and two RA facilitators. In total, this corresponds to $261 per classroom in the Enhanced condition. In-person facilitation accounted for 25% of our Enhanced costs; travel accounted for 23%; and incentives and resources accounted for 21%. The rest of the time was spent on preparation and miscellaneous tasks.

### Reach

#### Number of lessons delivered

Total compliance with returning WISE Material Reports was 86% for the school year and did not differ by condition. Based on these data, the average number of lessons delivered across all sites was 3.1 (SD = 0.9) per month with only December falling below a 3 average (*M* = 2.7, SD = 1.0); this was expected given the scheduled school winter break. This translated to an average reach across all sites of 70% (SD = 18.1). Average reach was higher in Enhanced classrooms (72% vs. 67%) and classrooms without turnover (73% vs. 66%), but not significantly so in between group comparisons (*t*_35_ = 0.73, *p* = .47 and *t*_35_ = 1.03, *p* = .31, respectively).

#### Number of resources distributed

The average number of weeks per month educators sent WISE handouts and/or recipes home to parents was 2.1 (SD = 1.4). All months, except for October (*M* = 1.9), November (*M* = 1.7), and December (*M* = 1.7), yielded an average of 2 or more weeks during which resources were sent. The total number of weeks in which resources were sent home did not significantly differ between the Enhanced classrooms (*M* = 13.4, SD = 7.8) and Basic classrooms (*M* = 14.6, SD = 7.3) sites (*t*_35_ = 0.49, *p* = .62).

### Effectiveness

#### Resonance Raman spectroscopy

A total of 320 children with parental permission participated in RRS at both the fall and spring assessments. This reflects 61% of the possible population of children receiving education at partnering sites. The average length of time between fall and spring assessments was 7.8 months (SD = 0.4). Increases in RRS values were significant from beginning to end of school year across all youth (*M*_diff_ = 3762.5, SD_diff_ = 7705.4) *t*_319_ = 8.8, *p* < .001) and for each treatment condition (Enhanced *M*_diff_ = 3327.0, SD_diff_ = 7822.0, *t*_158_ = 5.4, *p* < .001; Basic *M*_diff_ = 4192.6, SD_diff_ = 7588.2, *t*_160_ = 7.01, *p* < .001). A linear mixed model nested by classroom while controlling for fall RRS, child ethnicity, race, parental education, household food insecurity, and classroom turnover (i.e., one or both classroom teachers were replaced during the school year) found no statistically significant difference in RRS by treatment condition.

#### Body mass index-for-age percentile

Approximately 81% of youth had anthropometric measurements collected at both fall and spring, with an average of 6.2 months between assessment points (SD = 1.0). The majority of youth were within normal BMI percentile range at fall and spring (67.2% and 66.4%), with the overweight (17.3%, 16.8%) and obese (15.6%, 15.9%) categories reporting similar frequencies across time. Approximately 0.9% were in the underweight category at spring and none at fall. There was no significant change in BMI percentile across all youth (*M*_diff_ = 1.0, SD_diff_ = 20.7) *t*_421_ = 1.0, *p* = .33) nor within each treatment condition (Enhanced *M*_diff_ = 0.7, SD_diff_ = 20.7, *t*_203_ = 0.5, *p* = .62; Basic *M*_diff_ = 1.2, SD_diff_ = 20.7, *t*_217_ = 0.9, *p* = .38). A linear mixed model nested by classroom while controlling for fall BMI, ethnicity, race, parental education, household food insecurity, and classroom turnover found no statistically significant difference in BMI percentile by treatment condition.

### Adoption

#### Number of lessons prepared

On average, the agency purchased 82% of target servings to deliver a WISE lesson for each week that school was in session. This ranged from 46% of target servings for January (Carrot unit) to 112% for September and December (Apples and Bell Peppers, respectively). Differences between conditions cannot be computed due to cross service between sites. That is, the kitchen for some sites randomized to the Basic condition delivered to Enhanced sites and vice versa.

#### Organizational readiness for implementing change

The five items from the ORIC measure exhibited excellent internal consistency in the sample (Cronbach’s α = 0.96). Out of the possible scale of five, the observed mean for these items was 4.2 (SD = 0.9). In unadjusted between group comparisons, the Enhanced sites (*M* = 4.4, SD = 0.8) differed significantly from the Basic sites (*M* = 3.9, SD = 1.0; *t*_59_ = − 2.05, *p* = 0.045). An examination of the ORIC means by treatment condition demonstrated a higher mean (i.e., higher readiness) and tighter dispersion of scores for the Enhanced group (Supplementary File 2, Figure 3).

### Implementation

#### Fidelity

Unadjusted between-group comparisons demonstrated that the Enhanced group had significantly higher fidelity scores than the Basic implementation group for the EBPs of role modeling (*t*_34_ = − 2.5, *p* = 0.03), use of the mascot (*t*_34_ = − 4.0, *p* < .001), and hands-on exposure (*t*_34_ = − 2.5, *p* = 0.02) by the final data collection point despite non-significant differences at the first data collection point. Feeding practices did not differ between groups at any time point.

**Fig. 2:**
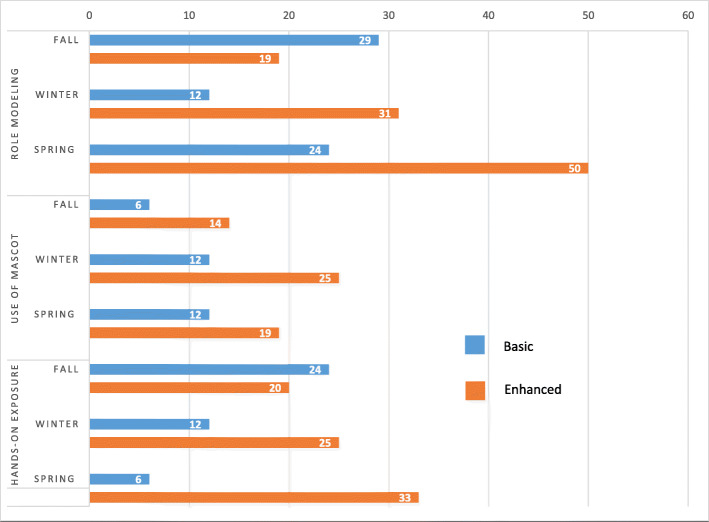
Percentage of classrooms achieving desired fidelity by quarter and treatment group

Figure 2 presents the percentage of classrooms by condition and across time that achieved a minimum desired level of fidelity (average score of 3 out of 4 for related items). For example, Basic classrooms began with 24% achieving fidelity for hands-on exposure at the fall assessment; this number fell to 6% by the spring assessment. Conversely, Enhanced classrooms began with 20% of classrooms achieving fidelity to hands-on exposure at the fall assessment and increased to 33% by the spring assessment. This resulted in a 27% difference in classrooms achieving fidelity between conditions at the spring assessment. Dispersion of fidelity scores for the 3 fidelity practices with univariate differences were explored while considering turnover in the classroom (See Supplementary File 2, Figures 3–5). For role modeling, the Enhanced group showed a higher mean as well as lower dispersion around the mean with several classrooms with turnover performing well at the final data collection. For mascot use, the Enhanced group showed a higher mean with a grouping of classrooms at the top-end of the scale; classrooms with turnover seem to perform better on role modeling in the Enhanced condition versus classrooms with turnover in the Basic condition. For hands-on exposure, the Enhanced group showed a higher mean; however, the classrooms with turnover did not have an advantage in the Enhanced condition.

#### Acceptability, appropriateness, feasibility

Internal consistency for each of the 3 adapted scales was excellent (Cronbach’s α = 0.98, 0.96, and 0.98, respectively). Means for the 3 scales indicated ratings consistent with an answer of slightly higher than “Agree” (*M*_Acceptability_ = 4.1, SD = 0.9; *M*_Aopropriateness_ = 4.1, SD = 0.9; *M*_Feasability_ = 4.2, SD = 0.8). For each of these outcomes, the Enhanced group showed a higher mean, and the Basic group consistently had educators reporting the lowest values. There are no notable patterns by classrooms experiencing turnover on these outcomes (See Supplementary File 2, Figures 6–8). Unadjusted mean comparisons demonstrated significant between group differences for Appropriateness (*t*_59_ = − 2.2, *p* = 0.03), but not Feasibility (*t*_59_ = − 1.9, *p* = 0.06) or Acceptability (*t*_59_ = − 1.8, *p* = 0.07).

#### Maintenance

Unadjusted between group comparisons showed no difference between Basic and Enhanced conditions for WISE fidelity for the lead educators that remained in the follow-up school year (Fall 2019, *N* = 19). An inadequate number of classrooms remained to conduct multivariate analyses. A summary of results for each domain of the RE-AIM framework is presented in Table 5.

**Table 5:** Formative interview themes related to participant-identified strengths and weaknesses of each implementation strategy

Implementation strategy	Weaknesses	Strengths
Formal Commitment Form	“I think any discussions that are held with site managers or whatever, the staff should be included so if they have questions at the very beginning, I think if those questions are answered, that they would buy into it a little bit better, and they wouldn’t be so I don’t know if “standoffish”. “I’m not sure what my commitment said.”	“And so, it was still being explained to me you know…what it was and making sure that I had an understanding of the form before I even signed it.”
Blueprint	None identified.	That [implementation blueprint] was very helpful to me and to the staff because I had to share this in the staff meeting because they would ask a question so, you know, they couldn’t remember what they did so I went and made copies of this. And gave everybody a copy. And we did have our training on this.
Cutting Board	“Well, to be honest, I seen that thing, but I didn’t know what it was for. And we had brought our own cutting board to use. Yea. We got it. But we didn’t know it was a cutting board. But we didn’t know it was a cutting board until about two weeks ago.”	“I love it. I use it for cutting the food, preparing the food on that. It’s so easy because you can just take it to the kitchen and get hot water and just you know wash it and wipe it off and re-use it again. It’s just nice….They said that it is Windy’s cutting board and you can’t touch it.”
Champion	None identified.	“On Wednesday, when we have our staff meetings, she’ll…She asks us how’s it going. She gives us some tips, ‘cause she gave us some good tips on, cause she said, the last thing she gave us was tips with what to do with our food experience. And she was talking about the small group time too, having them in small group. Which is help out, it does help out though with smaller group… She’ll come in here and ask me, you know, different tips on the WISE experience, what did we do, and what kind of activities did I do with the kids.”
Handouts (resources)	“I think the lady that delivered them came on a bad day. The day that she did it, we didn’t accept them very well. So, we just kind of picked them up and stuck them in the corner.” “Some of it I have [read]… but I have probably got it though because I get so many…”	“Also, and then I would know…. I can look at those objectives. And think about how it applies…”
Video (resources)	“I haven't been online…”	“Okay. Well, I mean, they were helpful looking at different things telling us how different classrooms or other people did things, and it was just informative. Like especially the one this morning I looked at. And I was like, you know, I put my food on the plate, you know, ‘cause we have to eat the same things with them. And it was just on the plate, and I’m talking to them and stuff, but every once in a while, they say, “___, are you gonna… you don’t like it?” you know. So really go ahead and eating, tasting, you know, ‘cause we want them to taste it, we gotta taste it. They were very appealing. There’s nothing I would change. They’re short enough, you know, enough time you can look at it and go on and grab the information. I think there was a part where I read and then the video came through.”
Incentives	“I don’t know who she gave it (bad report) to because she came back and she gave the other classroom a gift, an award, and a certificate, and didn’t give ______ nothing. Well you all didn’t do what you were supposed to do. We were like what were we supposed to do? The kids were engaged, we talked to them, and we encouraged them. They at the taste test, we did math with the apples, we did this and you still gave us a bad report. Oh no something is wrong with this picture...and the teachers don’t even know how they got them (incentives) because they said “We didn’t do nothing”.”	“I love it. Keep bringing it in, we need more stuff. [Unintelligible] we got so excited. And ____ got the stamp, the Windy Wise stamp, and when I took it over there to her, I was like “look what you got for using Windy!” She was like, “wow! You know it’s real, like…I don’t think the people expected they were going to get incentives or whatever. But I think they’re real nice, just keep them coming… So I’m pretty sure the other teachers are kind of grateful. I would just say grateful, but they’re probably glad that they are receiving something, because then they’re showing their efforts are being appreciated.”
Facilitation	“You have to get WISE in and you have to get this in. And then people coming in to observe you. It’s like the magnifying glass is on you. You know…and then they are going to find something wrong. You not doing this the right way. Then um you like…when the lady came in to us we were doing apples right? And I don’t know if we didn’t do it the right way, or we did something wrong, or whatever. She gave us a bad report. I feel like we did good because the kids were engaging and they were doing everything. They did the taste test and they cut, and they counted, and they did all of that and she gave us a bad report. So I don’t know if it was because we didn’t do the activity that she had planned on seeing from us I don’t know who she gave it to because she came back and she gave the other classroom a gift, an award, and a certificate, and didn’t give ____nothing. Well you all didn’t do what you were supposed to do. We were like what were we supposed to do? The kids were engaged, we talked to them, and we encouraged them. They ate the taste test, we did math with the apples, we did this and you still gave us a bad report. Oh no something is wrong with this picture.”	“They were pretty communicative. Yeah, they pretty much communicated with me about everything. If I asked for something, I think I asked for something one time, they brought it back over.” “Well, they give us good support. I can’t say that they don’t give us good support because they do. And when they come if we have any questions or anything, they are right here to answer it for us so that’s great. They are great resources and support for us.”
WISE innovation	“It says as a small group, I kind of like it better as a large group because I guess it's better for me. But like I'm going to try with the smaller group.” “Ooh, the hardest part, if it’s something that I don’t like… most times I put it towards my mouth, and [?] you know, chewing, like I’m really eating it and stuff like that. But most times, the stuff that we have, most times I try it.”	“Being able to taste the...well giving the children that experience, the tasting of fresh fruits and vegetables.” “The best thing so far is that we have to have cooking experience, we don’t have to come up with anything, we’re getting the supplies that we need for the cooking experience, and they’re healthy.” “And I think that once the children get used to having Windy to bring vegetables and fruits and if you all stop, then I think that besides at school they might not get it. So, I would say just keep on doing it because that may would encourage children to eat healthier and less obesity.” “Well the best thing that I figure with WISE is that the children get to experiment or participate in different types of fruit and vegetables that they may not eat at home. So that’s a good thing and introducing it to them. Some of them don’t even know what it is so…we introduce that fruit and vegetable to them and they get to taste it, feel it, and touch it. The parents don’t cook it or use it at home or make it.”
Context	“The worst thing is not getting the supplies on time. We do it on Tuesday’s. Mm-hmm. Or sometimes it comes after the time that we are supposed to have it… We just fill the time with something else, or we will go ahead and talk about that vegetable or fruit or whatever it is and we will just talk about it, but we won’t… we will do something small, but not the activity that is actually planned for that day…We don’t go back.” “The hardest thing for me was just getting started, being the only person, the only teacher in here. But that’s about it. It’s not really too hard this far. Like we have all the supplies, we have Windy Wise, and I like talking with the puppet with them. So, not too hard. Just having another person in here. When I did the experience, maybe someone else be in here with me. Sometimes I did have another TA like me, but just explaining and all that, it still kind of fell on me to do the whole thing.” “There’s no input from my site manager. There’s no input or like, coming to see if we, you know, just sitting in on the class to see what we’re doing or something, so… No that’s just it, being involved in the whole thing when we’re doing it, just to see how we’re doing and how the kids are enjoying it.” “We don’t really [talk about WISE]…well when it first got started we would talk about it. I think a lot of us were complaining about it, but we would just do it you know…kind of getting used to it now so.”	“She [director] will come in sometime. Well I don't know what she did in the rooms. But she will come in and she would actually sit down and join us for the experience or you know, and sometimes she would help because it was hard to do in small groups and she would kind of help micro manage stuff.” “We have teachers’ meetings on every Tuesday, and we talk about WISE. All of them like it. They are really fascinated over it.” “We [teachers] do it [talk about WISE] almost every day. Cause we, you know, we wanna, that way we know what we’re supposed to do for that day. And it helps us, it helps us, you know, along, cause that way she might be doing something different activity with her kids, and helping, you know, with my kids. You know, I may be doing something different I may help her with, but it’s not like every day.” “And she [director] reminds us and if we don't have the supplies we need, she do make sure we get them.”

### Interviews

Table 3 provides themes related to participant-identified strengths and weaknesses of each implementation strategy. These data were discussed with the EBQI panel and improvements to the strategies were based on the processing of data with the EBQI stakeholders when possible. For example, iterations were made during the school year (e.g., site choice about person to complete incentive delivery). Iterations for future use of the strategies will include purchasing higher quality cutting boards with WISE EBP reminders, more specific training on requested topics (e.g., working in small groups, teaching hunger cues), improved framing for incentive delivery, as well as better informing sites of the role of the Site Champion.

### Key events and departure from planned protocol

Several key events affected the process and outcomes of our study. Table 6 presents a catalogue of these events consistent with the key events template from Woodward et al. [60]. These key events provided important context for interpretation of study findings and for understanding changes to the study protocol. Because of these challenges, our partnering site lifted the requirement for end-of-year family assessments. These end-of-year assessments would have supplied the food frequency questionnaire data to provide an additional indicator of Effectiveness and were not included in the study as planned. In addition, the study team planned to use the ORCA instrument as our key indicator of Adoption. However, we gathered feedback on our instruments from stakeholders who felt ORCA items were cumbersome and difficult to answer. Stakeholders preferred items from the Organizational Readiness for Implementing Change (ORIC) instrument [52], primarily for reasons of clarity. We retained ORCA as a baseline assessment, which was completed anonymously by staff at a professional development day near the end of the prior school year and averaged across staff at the same site to obtain a site-level organizational assessment. We used ORCA to inform randomization and ORIC as an indicator of adoption. Additions to the published protocol included collection of the baseline survey to inform randomization, process evaluation of the delivery of the multi-faceted implementation strategy package, and estimation of costs for its delivery.

**Table 6:** Key events affecting study process and outcomes

Key Event category	Observed event
1. Personnel Changes	1.1. There was high turnover during the implementation year; 43% of teacher positions experienced turnover during the school year (range of 0 to 72% by site). This corresponded to 61% of classrooms affected by turnover (range of 0 to 100% by site). Turnover rates did not differ between the Basic and Enhanced groups. 1.2. Director layoffs between implementation and maintenance year; all directors were reassigned or their employment terminated between the 2 years.
2. Facilitator Activities	2.1. Facilitators met with each site’s leadership for onboarding, attended and supported the 6-h training on WISE, and provided coaching for the Enhanced condition in the implementation year. 2.2. A facilitator was trained and added mid-year when one of the two study facilitators required shoulder surgery.
3. Site Events	3.1. All teachers were required to change the location of their classroom between the implementation and maintenance year; most moved sites. 3.2. All classroom teacher pairs (lead and assistant) were changed between the implementation and maintenance year such that no teaching pair remained intact in the treatment group.
4. Broader Context Events	4.1 The organization experienced funding uncertainty beginning near the end of the implementation year. No teacher was assured the ability to return to a position for the following year (study maintenance assessment year). The organization was unable to communicate about funding to the staff until the start of the next school year. This uncertainty contributed to more staff turnover and layoffs, leaving only 19 of 75 teachers (25%) for maintenance assessment.

## Discussion

The stakeholder-selected strategies in this study demonstrated significant improvements on indicators of Adoption and Implementation. Specifically, the Enhanced group had significantly higher fidelity to 3 WISE EBPs, Organizational Readiness for Implementing Change, and perceived Appropriateness. Despite these indicators of promise, the multi-faceted strategy package did not translate to improved effectiveness outcomes for children within the school year timeline. In 2019, experts in implementation science recommended an agenda for advancing research on implementation strategies [61]. Our study design aligns with suggested priorities. First, our study combined approaches (concept mapping [33], evidence-based quality improvement [30], liberating structures [31]) to “enhance the design and tailoring of implementation strategies” that were tested in the Enhanced condition of our study. Our process is the first stakeholder-partnered initiative to build and test implementation strategies in early care and education. Second, we collected data on the costs of delivering the multi-faceted implementation strategy package. A focus on cost was added to provide information on the potential scalability of the multi-faceted strategy. This was possible because we deployed an app-based approach to “tracking and reporting of implementation strategies” that allowed us to provide detailed information on the dosage and content of the implementation strategies. That is, we adapted a system by Ritchie and colleagues [62] for tracking facilitation activities for field work to track delivery of all implementation strategies. Thus, our study illustrates best practices and advancements in implementation science studies, especially as applied in a community setting.

The improvements observed in the proportion of classrooms achieving fidelity ranged from 5 to 31% for our Enhanced group (for the 3 improved EBPs, see Fig. 2). Prior reviews [63–66] have reported on the median effectiveness of some of the discrete strategies that were included in our enhanced multi-faceted strategy package. For example, in healthcare settings, printed educational materials have accounted for a median of 4% improvement in healthcare professionals’ practices [63], the relative same proportion of practices improved by audit and feedback [64]. A review of educational outreach identified 5.6% median improvement [65], while local opinion leaders (akin to Champions in our study) have been linked with 11% improvement [66]. Further, a 2016 review of 10 implementation studies in childcare for healthy eating, physical activity, and obesity prevention practices, policies, and programs found between 0 and 9.5% implementation of targeted innovations [67]. These observations provide context for the interpretation of the improvements we observed (i.e., our fidelity increases exceeded those observed in these studies), which are unique from prior implementation work in the target of early care and education teachers as the implementers of interest for nutrition practices [26].

Consistent with prior work [12, 68], our study demonstrated positive effects of the WISE intervention on child intake of carotenoid rich foods. However, like the majority of other studies aimed at implementing practices and policies to improve child weight in the early education setting [67], no effect on BMI was observed. We believe the findings on child outcomes should be considered in tandem with findings on implementation fidelity. Specifically, educators in the Enhanced group increased in their fidelity to the intervention across the school year while educators in the Basic group remained the same or decreased in their fidelity resulting in the significant treatment difference. Thus, children in Enhanced classrooms were receiving a higher quality intervention experience near the end of the study (and time of outcome assessments), but their aggregate experience with the intervention across the school year was lower and nearer to the Basic group (see Fig. 2). BMI is a difficult metric to shift in community-based interventions given the complexity of settings in which families live, eat, and interact and the variety of factors that affect weight (e.g., diet, activity, stress, sleep) [69]. Therefore, it is not surprising that children in the Enhanced group, where educator improvement was largely in the last quarter of the year, did not differ from the Basic condition. Longer follow-up assessment periods may be required to see if improvements in fidelity translate to improvements in child outcomes.

This study includes both limitations and strengths. Foremost, there were a number of unforeseen and unusual circumstances for our partnering sites beyond the normal challenges of ECE that may limit the generalizability of our findings. Achieving these outcomes in light of key events of the study suggests promise for testing the multi-faceted strategy beyond the challenging circumstances of this sole agency and single school year. For example, the turnover documented in this study (43% of teacher positions overall, up to 72% per site) is notably higher than national estimates for Head Start turnover (17–20%) [70, 71]. Further, the uncertainty of the program’s funding and the news of coming layoffs and site/classroom moves near the end of the trial were disruptive. The research team adapted to these changes in delivery of implementation strategies (e.g., protocols to introduce WISE and facilitation to new educators), data collection (e.g., reducing planned maintenance analyses), and data interpretation (i.e., focus on univariate analyses and visual examination of data). The *t* test comparisons used as a result of these adaptations do not account for nesting in the data structure (i.e., teachers in sites), potentially leading to deflated standard errors and an increased chance of type I errors [69]. Finally, the Basic implementation group included only delivery of educational strategies (training and newsletters only), which may be seen as a less valuable comparison with a more active implementation strategy package. However, the content of the Basic implementation strategy was consistent with a recent review [26] documenting educational implementation strategies as those most commonly used in childcare-based implementation studies.

These limitations are offset by a number of strengths. This implementation effort was guided by an implementation framework (i-PARIHS) [72] that directed identification of key barriers and facilitators to use of the EBPs as well as collection of stakeholder feedback for the selection and tailoring of strategies, illustrating methods for theory-driven, community-engaged dissemination and implementation research. Further, we deployed a rigorous process evaluation, which will inform further iteration to the multi-faceted implementation strategy package. Formative interviews indicated key targets for improvement (e.g., improve explanation of site champion role to other teachers) as well as suggestions for exploration of the mechanisms of influence for these strategies (e.g., relationship with site champion). The PI was involved at each stage in the qualitative data collection and analyses, allowing for interpretation of findings to be evaluated alongside experience in the interviews. We also collected information on the costs of delivery of the strategy package, a gap in implementation studies of healthy eating, physical activity, and obesity studies in ECE to date [67].

Findings of this study suggest several areas for future research. First, the component of WISE fidelity, that was not improved by the implementation strategy, was feeding practices. Evidence-based feeding practices of WISE were in conflict with other behaviors and attitudes that were prevalent among educators (e.g., pressuring to eat more, comparing children’s eating). These contrasting behaviors can compete for use at WISE lessons and mealtimes suggesting the need for a focused effort on de-implementation of inappropriate practices to determine if this supports both a reduction in use of such practices and creates space for implementation of supportive feeding practices. Second, the multi-faceted strategy tested in this study may be too resource-intensive and/or unnecessary for all sites in large-scale delivery. An adaptive implementation approach [73] which includes critical decision points and tailoring variables to inform optimized delivery in the real world may be beneficial. Finally, this study illustrates some of the organizational challenges faced by the ECE setting. Specifically, general capacity (as an element of organizational readiness) includes an organization’s receptivity to change, the culture and climate of the organization, resource allocation, leadership support, day-to-day processes, and staff skills and expertise [74]. Greater focus on organizational factors, and general capacity in particular, may be needed to advance implementation efforts in ECE. That is, efforts to stabilize the workforce, support a positive work environment, and prepare leadership to balance competing priorities to lead change may be needed as a foundational strategy.

## Conclusion

Overall, the study achieved many objectives that will be relevant to the field. First, we established and refined the mobile approach for auditing fidelity to our implementation strategy. We have posted the link to our log for public access; this can serve as an example to future intervention studies for leveraging a common resource (REDCap) for documenting delivery of implementation strategies. Second, we applied a rigorous mixed methods approach to document the challenges faced by participating sites and our personnel, and our adaptations to meet these challenges can be standardized and replicated in future implementation efforts. This has the potential to improve ongoing dissemination of the WISE intervention as well as future implementation studies in the ECE environment. For example, our qualitative data suggest incentives be used with caution in this context. Finally, although this study was powered for examining nested, multivariate models for child outcomes, other models were under-powered to account for nesting at the site level. Future implementation studies of WISE should include a greater number of sites for improved power and may need to consider a longer follow up time for assessing child effects given that teacher improvement occurred late in the school year. In addition to providing support for our multi-faceted strategy on indicators of Adoption and Implementation, the comprehensive nature of our evaluation provides a model for other studies assessing implementation strategies in ECE settings.

## Supplementary Information

**Additional file 1.** General Linear Models for Educator and Classroom Level Outcomes.

**Additional file 2: Figure 3**. Organizational Readiness for Implementing Change: Means at Final Time Point by Treatment Condition. **Figure 4**. Role Modeling: Means at Final Time Point by Treatment Condition. **Figure 5**. Use of Mascot: Means at Final Time Point by Treatment Condition. **Figure 6**. Hands-on Exposure: Means at Final Time Point by Treatment Condition. **Figure 7**. Acceptability: Means at Final Time Point by Treatment Condition. **Figure 8**. Feasibility: Means at Final Time Point by Treatment Condition. **Figure 9**. Appropriateness: Means at Final Time Point by Treatment Condition.

## Data Availability

The datasets used and/or analyzed during the current study are available from the corresponding author on reasonable request.

## Abbreviations

AR: Arkansas
BMI: Body mass index
EBP: Evidence-based practice
EBQI: Evidence-based quality improvement
ECE: Early Care and Education
i-PARiHS: Integrated Promoting Action on Research Implementation in Health Services
IRB: Institutional Review Board
ORCA: Organizational Readiness to Change Assessment
ORIC: Organizational Readiness for Implementing Change
REDCap: Research Electronic Data Capture
RRS: Resonance Raman spectroscopy
WISE: We Inspire Smart Eating
VA QUERI: Veteran’s Affairs Quality Enhancement Research Initiative
