# Supplementary material for: Stakeholder selected strategies for obesity prevention in childcare: results from a small-scale cluster randomized hybrid type III trial
Source: Implement Sci. 2021 May 1;16:48. doi: 10.1186/s13012-021-01119-x (PMC8088574; doi:10.1186/s13012-021-01119-x)
Supplement: Supplementary file 1 — Additional file 1. General Linear Models for Educator and Classroom Level Outcomes. [file 13012_2021_1119_MOESM1_ESM.docx]

## **Supplementary File 1:**

## **General Linear Models for Educator and Classroom Level Outcomes**

## **Reach**

Number of Lessons Delivered. A general linear model controlling for classroom turnover, educator race, and educator experience found no statistically significant fixed effect estimate for treatment condition or other covariates for predicting number of lessons delivered.

**Adoption**

Number of Resources Distributed*.* A general linear model controlling for classroom turnover, educator race, and educator experience found no statistically significant fixed effect estimate for treatment condition or other covariates.

Organizational Readiness for Implementing Change. A general linear model with intervention status as a predictor and controlling for educator race and educator experience found no statistically significant fixed effect estimate for treatment condition or other covariates.

**Implementation**

Fidelity. A general linear model controlling for educator race, educator experience, classroom turnover, and the initial time point explained a significant amount of variance in both hands-on exposure and mascot use at the final time point [*R^2^* _Hands-on_=.29, *F*=3.25 (27), *p*=.02; *R^2^* _Mascot_= .50, *F*=4.54 (27), *p* =.003]. Significant predictors of hands-on exposure at the final time point included educator experience with newer educators (1- 10 years of experience) demonstrating lower fidelity than educators with over 20 years of experience (*t_27_*= -2.77, *p*=0.01); intervention status was not a significant predictor (*t_27_*=1.92, *p*=0.06) beyond other controls. Significant predictors of mascot use were intervention status and educator experience. The Enhanced group had higher fidelity than the Basic group (*t_27_*=2.92, *p*=0.007), and newer educators (1- 10 years of experience) demonstrated lower fidelity than educators with over 20 years of experience (*t_27_*= -2.27, *p*=0.02). The adjusted models did not explain a significant portion of variance in role modeling or feeding practices.

Acceptability, Appropriateness, Feasibility*.* A multivariate general linear model examining treatment effects and controlling for educator race and educator experience found no statistically significant fixed effect estimate for treatment condition or other covariates for the outcomes of Acceptability, Feasibility, or Appropriateness.
