# Supplementary material for: Stakeholder selected strategies for obesity prevention in childcare: results from a small-scale cluster randomized hybrid type III trial
Source: Implement Sci. 2021 May 1;16:48. doi: 10.1186/s13012-021-01119-x (PMC8088574; doi:10.1186/s13012-021-01119-x)
Supplement: Supplementary file 2 — Additional file 2: Figure 3. Organizational Readiness for Implementing Change: Means at Final Time Point by Treatment Condition. Figure 4. Role Modeling: Means at Final Time Point by Treatment Condition. Figure 5. Use of Mascot: Means at Final Time Point by Treatment Condition. Figure 6. Hands-on Exposure: Means at Final Time Point by Treatment Condition. Figure 7. Acceptability: Means at Final Time Point by Treatment Condition. Figure 8. Feasibility: Means at Final Time Point by Treatment Condition. Figure 9. Appropriateness: Means at Final Time Point by Treatment Condition. [file 13012_2021_1119_MOESM2_ESM.docx]

Supplementary File 1

Figure 3. Organizational Readiness for Implementing Change: Means at Final Time Point by Treatment Condition


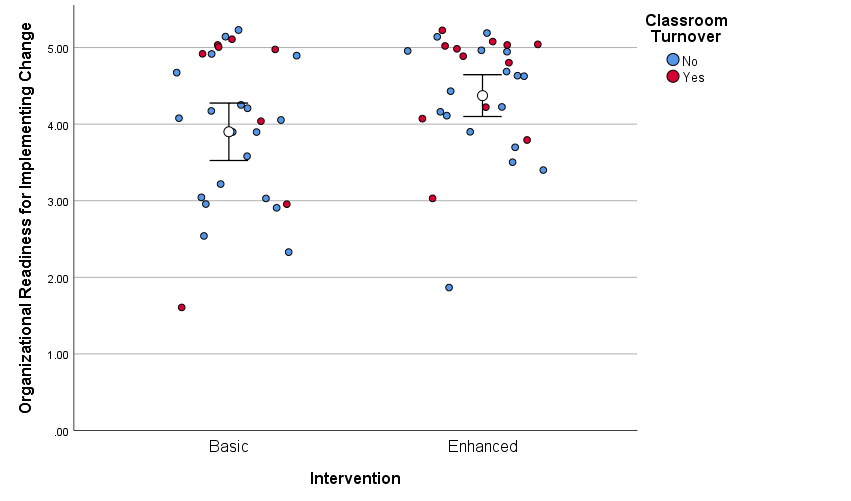


Notes: *The Basic implementation strategies involved training and reminders only; The Enhanced implementation strategy package including eight discrete implementation strategies, tailored to individual contexts and educators. Classroom turnover indicates whether either teacher was replaced during the school year.*

Figure 4. Role Modeling: Means at Final Time Point by Treatment Condition


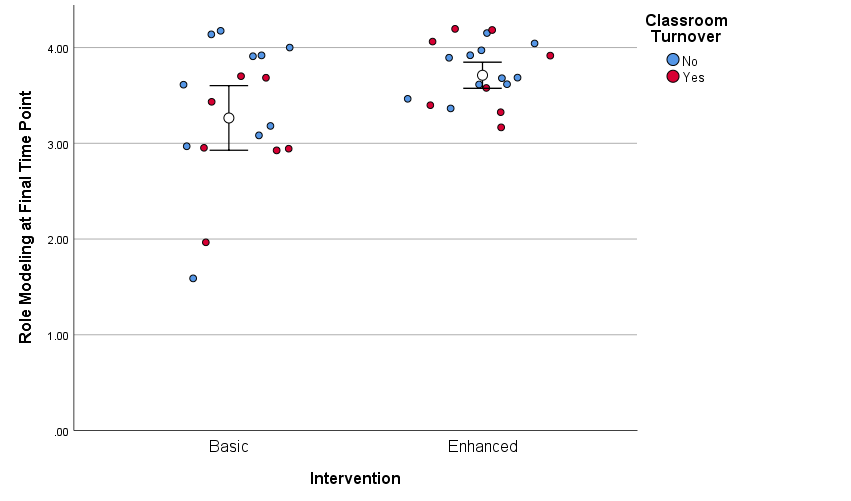


Notes: *The Basic implementation strategies involved training and reminders only; The Enhanced implementation strategy package including eight discrete implementation strategies, tailored to individual contexts and educators. Classroom turnover indicates whether either teacher was replaced during the school year.*

Figure 5. Use of Mascot: Means at Final Time Point by Treatment Condition


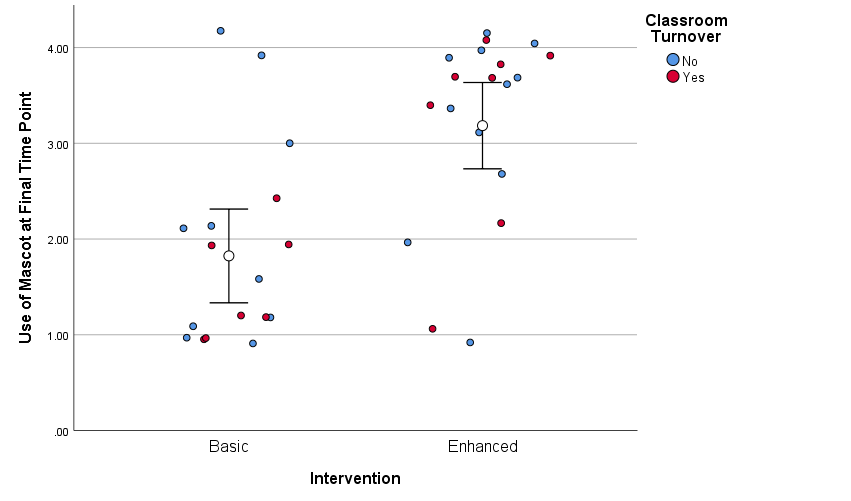


Notes: *The Basic implementation strategies involved training and reminders only; The Enhanced implementation strategy package including eight discrete implementation strategies, tailored to individual contexts and educators. Classroom turnover indicates whether either teacher was replaced during the school year.*

Figure 6. Hands-on Exposure: Means at Final Time Point by Treatment Condition


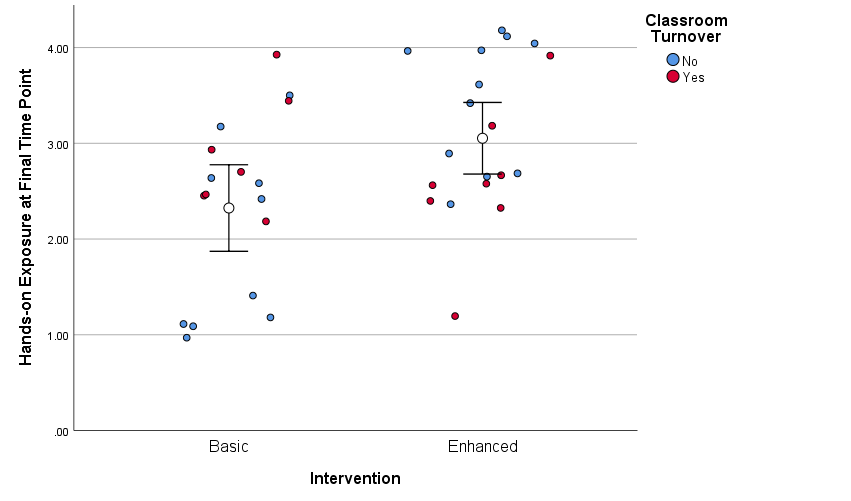


Notes: *The Basic implementation strategies involved training and reminders only; The Enhanced implementation strategy package including eight discrete implementation strategies, tailored to individual contexts and educators. Classroom turnover indicates whether either teacher was replaced during the school year.*

*.*

Figure 7. Acceptability: Means at Final Time Point by Treatment Condition


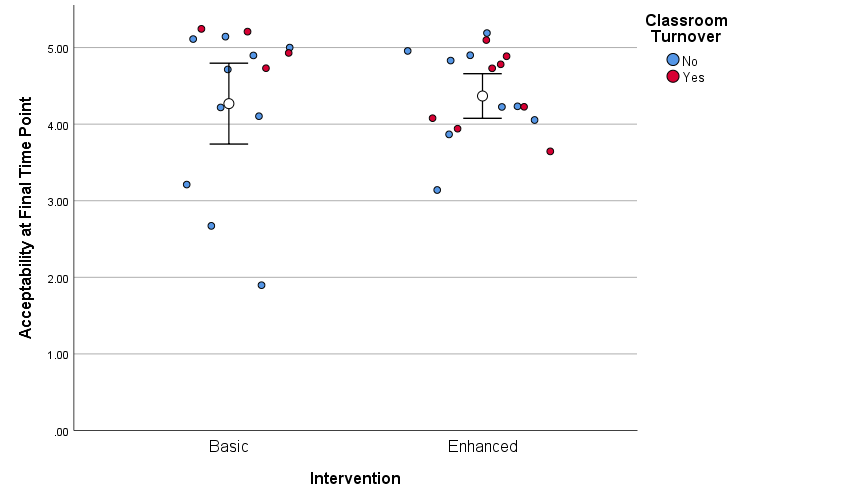


Notes: *The Basic implementation strategies involved training and reminders only; The Enhanced implementation strategy package including eight discrete implementation strategies, tailored to individual contexts and educators. Classroom turnover indicates whether either teacher was replaced during the school year.*

Figure 8. Feasibility: Means at Final Time Point by Treatment Condition


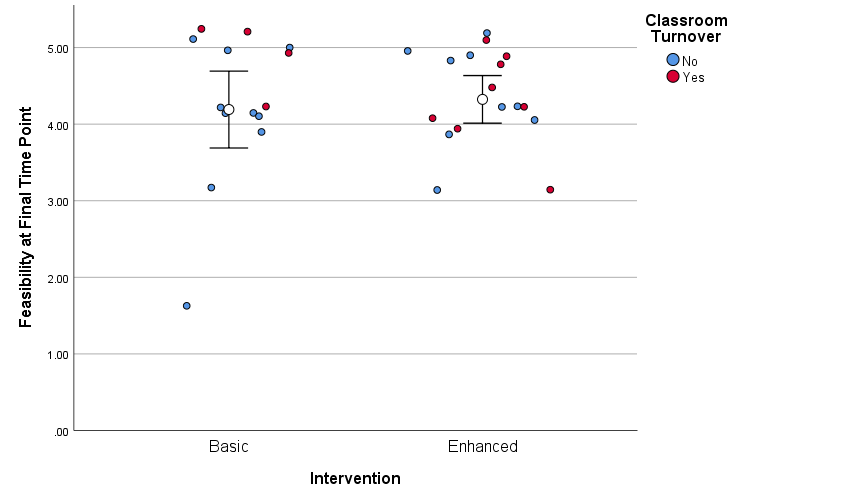


Notes: *The Basic implementation strategies involved training and reminders only; The Enhanced implementation strategy package including eight discrete implementation strategies, tailored to individual contexts and educators. Classroom turnover indicates whether either teacher was replaced during the school year.*

Figure 9. Appropriateness Means at Final Time Point by Treatment Condition


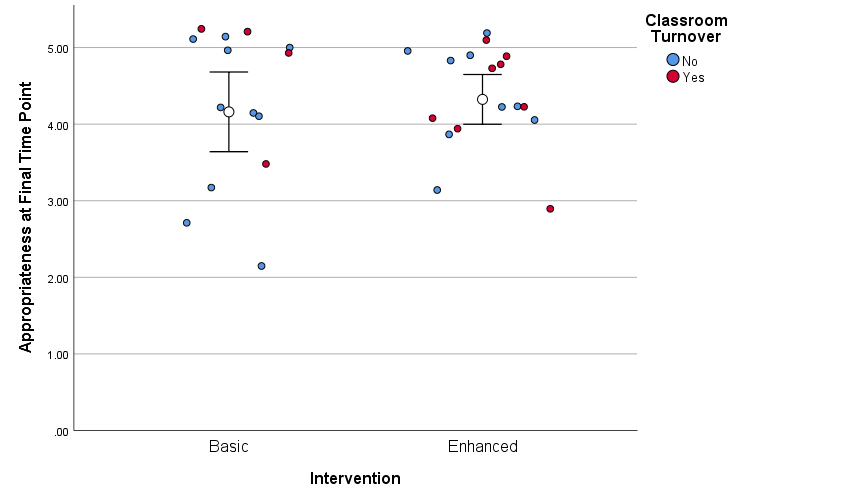


Notes: *The Basic implementation strategies involved training and reminders only; The Enhanced implementation strategy package including eight discrete implementation strategies, tailored to individual contexts and educators. Classroom turnover indicates whether either teacher was replaced during the school year.*
